# Sudan's armed rivalry: A comment on the vulnerable healthcare system catastrophe

**DOI:** 10.1002/hsr2.1517

**Published:** 2023-08-22

**Authors:** Lina Hemmeda, Alaa S. Ahmed, Maram Omer

**Affiliations:** ^1^ Faculty of Medicine University of Khartoum Khartoum Sudan

**Keywords:** global health, healthcare management, health services and outcomes research

## Abstract

The Sudan War 2023 is a military conflict that erupted in the heart of a paralyzed state system. A total of 68% of hospitals are presently closed, with the remainder operating just partially owing to a lack of staff, medical supplies, and pharmaceuticals. Furthermore, due to power outages, Sudan's vaccination and insulin stocks are under jeopardy. Additionally, patients with chronic conditions have difficulty receiving their prescriptions. This unaccounted‐for conflict is expected to degrade Sudan's already‐weak healthcare sector by the recent COVID‐19 pandemic and 2018 revolution. Humanitarian emergency care is being offered by numerous non‐for‐profit organizations and civilians initiatives; but, more measures, notably aimed at preventing political upheaval, are required to save the country's future.

## 1 INTRODUCTION

The Sudan War of 2023 is an armed battle between the Sudanese Armed Forces (SAF) and the rapid support forces (RSF), two factions of the country's military government. It erupted in Khartoum on April 15, 2023, and at least 12 other states were impacted by it. In particular, Merowe (City) in Northern States, El Geneina (City) in West Darfur, and El Obeid (City) in North Kurdufan.^1^ In light of this, the 2023 Sudan war should be viewed as a disastrous urban welfare with millions caught in the falling urban communities.^2^ The United Nations (UN) reported that more than 330,000 people are internally displaced within Sudanese states, and about 800,000 escaped to neighboring South Sudan, Chad, Egypt, and Ethiopia.^3^ Sudan's capital, Khartoum, currently the epicenter of this turmoil and conflict, is the most crowded state, hosting approximately 19% of Sudan's total population^4^ and providing the majority of Sudan's health services, more than third of the health provision^5^ due to the unfortunate centralized distribution. Dead bodies of both civilians and militants are scattered on the streets, and the civil servants are warning of an impending environmental disaster.^6^ The occurrence of armed fighting on the city streets has made public transportation exceedingly life‐threatening for the city residents. Plenty of death cases reported refer to citizens who had to leave their homes, aiming to buy groceries, to seek drinkable water or to seek medical help. These citizens had been killed either by stray bullets or by direct intended bullet attributed to suspicion. Furthermore, Khartoum and the other states have witnessed a severe economic standstill owing to the blocking of the internal trade routes. This has resulted in the unavailability of goods, safe water, pharmaceuticals, and fuels.^7^ This article sheds light on the present crisis in Sudan with the main purpose of pointing up to the level of deterioration that might occur to the Sudanese healthcare system in the event of a political crisis within the short timeframe of this article's reporting. By acknowledging this, we can bring situational awareness to decision‐makers and global influencers to prevent this from evolving into an impending catastrophe. Additionally, it is important to preserve historical records of the war timely and consecutively, which can in turn be used for further research and formal recording.

Before the ensuing war, the health system in Sudan had been seriously fragile, and minimal government investment had been put into healthcare infrastructure even before the latest political crisis. Following the start of the December 2018 Revolution in Sudan, the political instability, the subsequent military revolutions, the alternating health ministers with the absence of reform plans, as well as the stagnant economy, challenged the evolution of the healthcare system even after the COVID‐19 pandemic revealed many of the health system's insufficiencies.^8^ Inevitably, all these factors led to the vulnerability of the healthcare system and a “total collapse” shortly after the current conflict commenced,^5^ which could last for decades.

## 2 IMPACT OF WAR: HEALTHCARE SYSTEM IN DISARRAY

Due to the outrageous violence between SAF and RSF over the past few days, not only human lives are endangered, but Sudan's already ailing healthcare system is also rapidly collapsing. According to the preliminary committee of the Sudanese Doctors Union, until May 7, 2023, 68% of the hospitals adjacent to the conflict areas were out of service. Out of 88 hospitals in the capital and the affected states, 60 are suspended from service, and 28 are fully or partially operating; some of them provide only first aid services, and they're also threatened with closure due to a lack of medical staff, medical supplies, water, and electric current.^9^ According to the World Health Organization, just 16% of health facilities in Khartoum are now operational, with 61% shuttered^10^ owing to direct forcible evacuation and seizure by armed troops,^11^ shortages in medical supplies, or the inability of healthcare personnel to reach the hospitals as the streets have become a battlefield for the two rivals.^12^ Moreover, some hospitals are on the verge of closing down as a result of the prevalent looting or the unstable water and electrical supplies.^11^ In addition, over 40 million of Sudan's supplies of vaccination and insulin are at risk due to the inability to refuel generators.^12^ In El Geneina city, all medical facilities are inoperable due to attacks, looting, and assaults, as well as the looting of the central drug outlets, doctor's dwellings, and red crescent offices. Likewise, El Geneina's only renal center is currently out of service.^9^

According to the Federal Ministry of Health, the total number of casualties from the start of hostilities in April 15 till May 1 is 550, with 12 being healthcare staff and health sciences students and five being aid workers.^13^ As well, as of May 4, 2023, the United Nations International Children's Fund (UNICEF) reported at least 190 children murdered and more than 1700 wounded.^14^ In addition, a special consideration is given to people with chronic illnesses, who require a sustainable ongoing budget for payment as well as safe access to medical care, an original principle of universal health coverage. Furthermore, nearly 219,000 women will give birth without safe access to maternal care.^12^ Likewise, the UN warned that nearly 40% of the Sudanese population is at risk of impending food insecurity due to the war.^15^

A ceasefire has been proclaimed several times in recent days, but neither faction has entirely adhered to it. The population is now immobilized, with no access to basic necessities, let alone competent medical treatment. What makes the situation even worse for some people is their inability to travel overseas owing to airport closures around the country.^2^ The fighting has left the country in a state of chaos, and civilians are dying not only from airstrikes and stray bullets but also from robberies, as was the case when Dr. Bushra Suliman, a gastroenterologist who was revered as a mentor and the cofounder of the Sudanese American Medical Association, was shot and killed outside his home.

## 3 COORDINATING ACTORS AND RESOURCES IN A REVIVAL ATTEMPTS

Many health and aid organizations have stated that the conflict threatens to become a humanitarian disaster,^16^ with several expressing their willingness to provide support, but there are no safe corridors to allow aid to pass through; even if the corridors were secured for medical personnel and supplies, the situation is still catastrophic and urgently requires outside help. Doctors without borders (Medecins Sans Frontieres [MSF]) were able to donate medical supplies to Khartoum's health facilities. Additionally, they provided medical assistance to over 404 wounded patients in the El‐Fashir MSF‐supported hospital. However, as noted by MSF's North Darfur project coordinator, staff are unable to report for work while medical supplies are running low. Also, MSF declared recently that gunmen broke into their offices in Nyala, Darfur state, where they maintain essential medical equipment, and took everything, including cars and office supplies.^17^

The situation is “extremely complex,” as per the president of the International Federation of Red Cross and Red Crescent Societies (IFRC) office in Sudan. IFRC groups, in collaboration with the Sudan Red Crescent Society, remained active in 16 Sudanese states, as well as in border regions in Egypt, Chad, and Ethiopia, to provide basic well‐being services to the displaced. They focus on first aid and psychological support. Additionally, they both worked to launch an emergency appeal for Sudan for 30 million Swiss francs.^18^ On April 30, the United States Agency for International Development declared the deployment of a disaster response team that will be working from Kenya.^19^ UNICEF has also advocated for a system of connection to the electricity supply and fuel for backup generators for the cold chain.^20^

In furtherance, one of the unrecognized efforts is that the coordination performed by civilians who banded together on various social media platforms. They set up offers of assistance for those in need of food or medicine on Facebook, WhatsApp groups, and Twitter using Arabic hashtags like “#Khartoum_Needs.” They also provided information on safe routes out of the city for anyone in need of fleeing. Much of this aid is spearheaded by young volunteers who work on “resistance committees” at the local neighborhood level. These committees were founded as armours during the 2018 uprisings with the purpose of coordinating peaceful protests, and hundreds of them are currently in operation around the country. Likewise, local entities and associations of Sudanese physicians overseas are disseminating the names of volunteer professionals offering to conduct remote medical consultations by phone or through social media platforms as a global effort among immigrants devoted to averting the impending humanitarian disaster. With thousands of Sudanese escaping conflict zones, focus has switched to the creation and funding of specific community centers for displaced individuals at evacuation ports.^21^

## 4 CONCLUSION

Sudan's future is clouded by the present escalation of the conflict. The healthcare system is being overburdened by the conflict, which is destroying its infrastructure, claiming the lives of its employees, and leaving patients in need of assistance. Many humanitarian groups are attempting to assist. However, the poor urban welfare appears to outweigh their abilities.

## AUTHOR CONTRIBUTIONS

**Lina Hemmeda**: Conceptualization; methodology; project administration; writing—original draft; writing—review and editing. **Alaa S. Ahmed**: Conceptualization; methodology; writing—original draft; writing—review and editing. **Maram Omer**: Conceptualization; methodology; writing—original draft; writing—review and editing.

## CONFLICT OF INTEREST STATEMENT

The authors declare no conflict of interest.

## TRANSPARENCY STATEMENT

The lead author Lina Hemmeda affirms that this manuscript is an honest, accurate, and transparent account of the study being reported; that no important aspects of the study have been omitted; and that any discrepancies from the study as planned (and, if relevant, registered) have been explained.

## Data Availability

All data relevant to the study are included in the article.
